# Transparency and reporting characteristics of COVID-19 randomized controlled trials

**DOI:** 10.1186/s12916-022-02567-y

**Published:** 2022-09-26

**Authors:** Philipp Kapp, Laura Esmail, Lina Ghosn, Philippe Ravaud, Isabelle Boutron

**Affiliations:** 1 Université Paris Cité, Inserm, INRAE, Centre of Research in Epidemiology and Statistics (CRESS), F-75004 Paris, France; 2 Centre d’Épidémiologie Clinique, AP-HP, Hôpital Hôtel-Dieu, F-75004 Paris, France; 3 Cochrane France, F-75004 Paris, France; 4 Institute for Evidence in Medicine, Medical Center - University of Freiburg, Faculty of Medicine, University of Freiburg, D-79110 Freiburg, Germany

**Keywords:** COVID-19, Randomized controlled trial, Selective outcome reporting, Selection bias, Quality of reporting, Peer review; Completeness of reporting, CONSORT, Transparency

## Abstract

**Background:**

In the context of the COVID-19 pandemic, randomized controlled trials (RCTs) are essential to support clinical decision-making. We aimed (1) to assess and compare the reporting characteristics of RCTs between preprints and peer-reviewed publications and (2) to assess whether reporting improves after the peer review process for all preprints subsequently published in peer-reviewed journals.

**Methods:**

We searched the Cochrane COVID-19 Study Register and L·OVE COVID-19 platform to identify all reports of RCTs assessing pharmacological treatments of COVID-19, up to May 2021. We extracted indicators of transparency (e.g., trial registration, data sharing intentions) and assessed the completeness of reporting (i.e., some important CONSORT items, conflict of interest, ethical approval) using a standardized data extraction form. We also identified paired reports published in preprint and peer-reviewed publications.

**Results:**

We identified 251 trial reports: 121 (48%) were first published in peer-reviewed journals, and 130 (52%) were first published as preprints. Transparency was poor. About half of trials were prospectively registered (*n* = 140, 56%); 38% (*n* = 95) made their full protocols available, and 29% (*n* = 72) provided access to their statistical analysis plan report. A data sharing statement was reported in 68% (*n* = 170) of the reports of which 91% stated their willingness to share. Completeness of reporting was low: only 32% (*n* = 81) of trials completely defined the pre-specified primary outcome measures; 57% (*n* = 143) reported the process of allocation concealment. Overall, 51% (*n* = 127) adequately reported the results for the primary outcomes while only 14% (*n* = 36) of trials adequately described harms. Primary outcome(s) reported in trial registries and published reports were inconsistent in 49% (*n* = 104) of trials; of them, only 15% (*n* = 16) disclosed outcome switching in the report. There were no major differences between preprints and peer-reviewed publications. Of the 130 RCTs published as preprints, 78 were subsequently published in a peer-reviewed journal. There was no major improvement after the journal peer review process for most items.

**Conclusions:**

Transparency, completeness, and consistency of reporting of COVID-19 clinical trials were insufficient both in preprints and peer-reviewed publications. A comparison of paired reports published in preprint and peer-reviewed publication did not indicate major improvement.

**Supplementary Information:**

The online version contains supplementary material available at 10.1186/s12916-022-02567-y.

## Background

In response to the global COVID-19 pandemic, clinical research has accelerated dramatically. In April 2021, about 2900 randomized controlled trials (RCTs) on interventions for COVID-19 have been registered [1–3]. Overall, it was estimated that more than 20,000 published articles have been indexed on the Web of Science and Scopus between January and June 2020 [4].

The communication of scientific results has considerably evolved to respond to the need and request for rapid information from policymakers, guideline developers, health care providers, and the public [5]. Some journals reacted by accelerating considerably their editorial processes to ensure that clinically actionable information was rapidly made available [5–7]. However, this speed in the process also had some drawbacks as it could reduce the rigor of manuscripts’ evaluation. Indeed, concerns were raised about the quality of published results [8, 9]. Some of these concerns resulted in retractions of high-profile and impactful publications [9].

In this context, it is important to implement safeguards to protect research integrity and transparency. Access to all trial documentation and adherence to reporting standards such as the Consolidated Standards of Reporting Trials (CONSORT) statement are particularly important to reduce research waste and improve research reproducibility [10, 11]. Nevertheless, some studies evaluating adherence to CONSORT statements at an early stage of the pandemic and on a limited sample raised some concerns about the completeness of reporting [12].

The COVID-19 pandemic was also associated with a considerable increase in communication of results through preprints [13–16]. Preprints are manuscripts shared through an open-access preprint server. The manuscript has not been peer-reviewed. Some preprint servers required the reporting of specific information and make very simple checks before making the manuscript public [17]. Preprint servers offer the possibility to disseminate research results earlier compared to the usual journal editorial processes. The authors can gain better and wider feedback from the communities who can comment on preprints. It is also an opportunity for the authors to give open access to a version of their scholarship.

During the pandemic, preprints were more frequently used and were more likely to be published in a peer-reviewed journal with a very short delay [13, 16]. Some preprint servers gained up to 25% more trials due to COVID-19 [13]. Nevertheless, concerns have been raised about the quality of preprints and on the consistency between the preprint and the related peer-reviewed publication [18–20].

To explore the scholarly communication of COVID-19 trials’ results in more depth, we conducted a systematic review to assess (1) the transparency, completeness, and consistency of reporting in reports of RCTs assessing pharmacologic treatments for COVID-19 and (2) the impact of the journal peer review process on reporting and transparency for all preprints subsequently published in peer-reviewed journals.

## Methods

### Protocol

This study is part of the COVID-NMA initiative (PROSPERO CRD42020182600) [1, 2, 21]. The two first pillars of this initiative are a living mapping and living evidence synthesis of all randomized controlled trials assessing treatments and preventive interventions for COVID-19. All results are updated weekly and made available on an open access platform (https://covid-nma.com) [1, 2, 21].

The third pillar of this initiative, which is described in this manuscript, is the monitoring of trial reports in terms of transparency and reporting (protocol on Zenodo: 10.5281/zenodo.5810076) [1]. Because of the context and resource constraints, the scope was reduced to trials of pharmacological treatments and to the assessment of transparency, completeness, and consistency of reporting. Because of the role of preprint in scholarly communication, we added the comparison between preprint and related peer-reviewed publication.

### Study design

We conducted a systematic review of randomized controlled trials published for the treatments of COVID-19 up to May 31, 2021.

### Eligibility criteria

We included RCTs assessing pharmacological treatments such as antivirals, interferons, other antimicrobials, non-steroidal anti-inflammatory drugs, vitamins, kinase inhibitors, corticosteroids, monoclonal antibodies, immunosuppressants, antithrombotic but also convalescent plasma, and advanced therapy medicinal products (ATMP).

Trials assessing non-pharmacological interventions (e.g., prone positioning, physiotherapy), pharmacological treatment of long-COVID, and preventive interventions, including vaccines, were excluded. Studies that did not randomly allocate patients to a treatment arm (e.g., quasi-randomized studies, phase one trials, single-arm trials) and modeling studies of interventions for COVID-19 were also excluded. We included trials published as research articles (i.e., full report), while other publication formats (e.g., conference abstracts or comments) were excluded. We only included trials written in English.

### Search strategy

The search strategy was developed in collaboration with an information specialist from the Cochrane Editorial & Methods Department as part of a living systematic review.

The search strategy evolved over time and relied on two high-quality secondary sources: the Epistemonikos L·OVE COVID-19 platform (app.iloveevidence.com/covid19) [22] and the Cochrane COVID-19 Study Register (covid-19.cochrane.org/). We also searched the Retraction Watch Database for retracted studies (retractionwatch.com/retracted-coronavirus-covid-19-papers).

The search strategy and data sources are detailed in the Additional file 1: Table S1. The last search was conducted on May 31, 2021.

Two reviewers independently screened all retrieved titles and abstracts in duplicate using Rayyan [23]. Discrepancies were resolved by consensus between the two reviewers. A third reviewer was involved to resolve disagreements when necessary.

### Paired identification for preprint-related peer-reviewed publication

The search allowed the identification of both preprints and peer-reviewed publications.

For all trials published first as a preprint, we systematically searched for a subsequent publication in peer-reviewed journals using a preprint tracker on a weekly basis (https://dbrech.irit.fr/pls/apex/f?p=104:3—last search October 7, 2021) [24]. We entered the preprint DOI, preprint venue, and preprint data. The tracker provides a list of relevant publications. The candidate list of preprint-publication pairs is sorted by decreasing the likelihood of preprint-publication association. One reviewer screens all of the pairs and identifies the publication reporting the related trial results.

### Data extraction

We specifically developed a standardized, online data extraction form covering general trial characteristics, transparency indicators, completeness, and consistency of reporting on the COVID-NMA platform. For reports published as a preprint and a peer-reviewed journal publication, we assessed the first publicly available report.

To reduce errors during the extraction and ensure calibration, two reviewers were trained and separately assessed 20 trials each through oral and written instructions. The reviewers discussed the meaning of each assessment item and reached a consensus for the 20 trials. Subsequently, all included trials were extracted by a single reviewer. The inter-rater agreement between the two reviewers was good with 96.6% agreement, with a kappa coefficient of 0.87 (95% CI, 0.83–0.92).

#### General characteristics of the trials

We extracted the trial design, number of arms, sample size, setting, number of centers, blinding, type of publication (preprint, journal peer-reviewed), subsequent publication of preprint studies, and funding sources (i.e., private through industry sources or public, which involve primarily governmental funds). We also extracted the type of treatments, the setting (hospitalized vs outpatient ambulatory care), and the severity of the disease of the included participants [25].

#### Transparency indicators

Transparency indicators refer to accessible sources of information such as the protocol, the registry, and the statistical analysis plan that are essential for the comprehension of what was planned and performed. We considered the following indicators of transparency:

1. *Access to the trial documentation*: We checked whether we had access to the protocol and statistical analysis plan and if it was available in English.
2. *Trial registration*: We evaluated whether trials were registered by using the registration number reported in the manuscript or associated documents. If none was reported, the study is classified as not registered unless we obtained the registration number through other sources (e.g., contact of authors). If registration was done prospectively (i.e., before the initiation of recruitment) and if trial results were posted when the registry had a specific field for the investigator to report the trial results, the following primary registries had this option available: ClinicalTrials.gov, EU Clinical Trial Register, ISRCTN registry, DRKS – German Clinical Trials Register, jRCT – Japan Registry of Clinical Trials, and ANZCTR – Australian New Zealand Clinical Trial registry. The reference to the published report in the registry was not considered as posted result.
3. *Data sharing statement*: We searched in the report, its appendix, and in the online version of the report for a data sharing statement, i.e., a statement provided by the authors indicating whether, how, and when they are sharing the individual participant data. For the corresponding trial registry, we retrieved information from the dedicated data sharing section, if available. We considered any kind of data sharing statement, without restrictions on the type of data sharing (e.g., on email request, online repository). We extracted the type of data sharing.

#### Completeness of reporting

We systematically evaluated whether the trial report and protocol, if available, adhered to the Consolidated Standards of Reporting Trials (CONSORT) 2010 statement [11, 26]. We decided to focus on 10 CONSORT items which were deemed most important because they are frequently incompletely reported and are necessary for conducting a systematic review, to evaluate the risk of bias and record the outcome data [27]. The completeness of reporting was assessed using the COBPeer tool (in Additional file 1: Table S2) [27]. For each item extracted, the COBPeer tool evaluates the CONSORT items and associated sub-items and generates what should be reported as stated in the CONSORT 2010 Explanation and Elaboration Explanation paper [11, 27]. Reviewers had to indicate if the requested information was reported for each sub-item (yes/no). Finally, each item was rated as “completely reported” if all sub-items were adequately reported, “partially reported” if at least one sub-item was missing, and “not reported” if all items were missing. For the assessment of the CONSORT items, we systematically considered the primary outcome of the report. If the primary outcome was not clearly identified, we considered the outcome reported in the objective, and if none was reported, we assessed the completeness of reporting of all outcomes reported in the publication and recorded the least adequately reported.

In addition to CONSORT-related items, we assessed if the authors reported information on funding, conflicts of interest for the primary investigators and trial statistician, and ethical approval.

#### Consistency of reporting (i.e., primary outcome switching)

We assessed the first publicly available report for consistency between what was planned and reported in the registry and what was reported in the publication. Particularly, we checked for primary outcome switching between the registry and the report. Primary outcome switching was defined as adding, removing, or changing a primary outcome (i.e., the variable of interest, time frame, or metric). Trials that failed to provide any timing information in the report or trial registration were assessed only for a change in the variable of interest.

For the assessment of outcome switching, all available registration platforms were used. If the trial registration was modified after the study start date, we considered the latest registration entry before the trial started, if available. We checked whether outcome switching was disclosed in the report. Explanations and justifications were considered as valid, as soon as the authors indicated the changed primary outcomes in the report (e.g., in the introduction or discussion sections of the report).

#### Comparison between preprint reports and related peer-reviewed journal publication

For preprints subsequently published in a peer-reviewed journal, we compared the reporting of the first publicly available preprint report available to the peer-reviewed publication. Changes between the preprint and the peer-reviewed journal publication were classified as “added” information (i.e., information missing in the preprint report but reported in the publication) or “removed” information (i.e., information reported in the preprint report but removed in the publication) [28]. In addition, we assessed if primary outcome switching changed between the preprint and the peer-reviewed journal publications.

### Data analysis

The descriptive analysis consisted of frequencies, percentages, and medians with interquartile range. We also report the absolute risk difference and 95% confidence interval (using the Wald method) to compare the reporting between preprint and subsequent peer-reviewed publications.

## Results

### RCT identification and characteristics

**Fig. 1:**
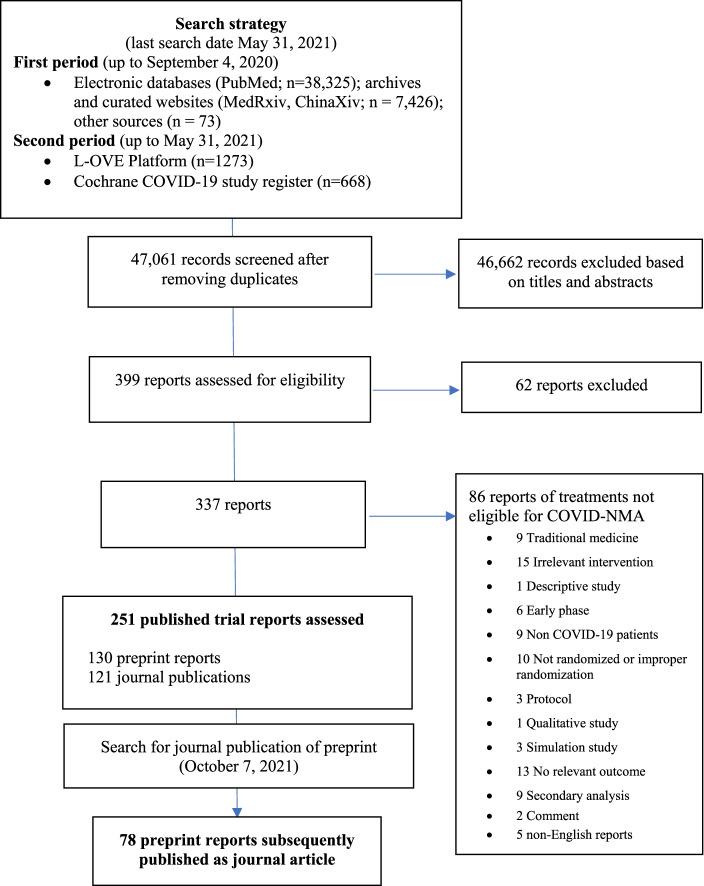
Flowchart of the included randomized controlled trials (RCTs) (last search date May 31, 2021)

The results of the search are detailed in Fig. 1. Of the 47,061 records screened, 251 reports of randomized trials evaluating pharmacological treatments of COVID-19 were identified and assessed. Overall, 121 (48%) RCTs were first and only published in peer-reviewed journals while 130 (52%) were initially available as preprints. Of the 130 preprints, 78 (60%) were subsequently published in a peer-reviewed journal.

Table 1 provides information on the general characteristics of the 251 trials. Overall, 89% (*n* = 223) were conducted in a single country (countries with the most conducted trials were Iran [*n* = 40], China [*n* = 31], the USA [*n* = 26], and Brazil [*n* = 20]). Most RCTs used a 2-arm design (*n* = 216, 86%). Patients were mainly hospitalized (*n* = 204, 81%). The most common study treatments were antimicrobials (*n* = 52), antivirals (*n* = 50), other monoclonal antibodies (*n* = 28), convalescent plasma (*n* = 17), and corticosteroids (*n* = 11). The median sample size was 101 (IQR: 56–253) (range: 10 to 11,558). Less than half of the trials were funded by public sources (*n* = 101, 40%), 64 (18%) received mixed funding (public and private), while 46 (18%) received solely private funding. Twenty-seven reported no specific funding (11%), and 13 trials did not provide any funding information.

**Table 1:** Randomized trials characteristics

Characteristic	Overall, ***n*** = 251	Preprints, ***n*** = 130	Journal publications, ***n*** = 121
**Number of arms**			
2 arms	216 (86%)	111 (85%)	105 (87%)
More than 2 arms	35 (14%)	19 (15%)	16 (13%)
**Sample size (median, IQR)**	101 (56–253)^a^	100 (56–268)^a^	103 (60–237)
**Setting**			
Single country^b^	223 (89%)	112 (86%)	111 (92%)
Iran	40 (16%)	19 (15%)	21 (17%)
China	31 (12%)	11 (9%)	20 (17%)
USA	26 (10%)	11 (9%)	15 (12%)
Brazil	20 (8%)	7 (5%)	13 (11%)
India	20 (8%)	12 (9%)	8 (7%)
UK	11 (4%)	8 (6%)	3 (3%)
Egypt	10 (4%)	5 (4%)	5 (4%)
Argentina	7 (3%)	6 (5%)	1 (1%)
Mexico	6 (2%)	5 (4%)	1 (1%)
Bangladesh	5 (2%)	2 (2%)	3 (3%)
Pakistan	5 (2%)	2 (2%)	3 (3%)
Spain	5 (2%)	3 (2%)	2 (2%)
France	3 (1%)	0 (0%)	3 (3%)
Netherlands	3 (1%)	3 (2%)	0 (0%)
Multinational	28 (11%)	18 (14%)	10 (8%)
**Number of centers**			
Single-center	104 (41%)	53 (41%)	51 (42%)
Multicenter	144 (57%)	77 (59%)	67 (55%)
No information	3 (1%)	0 (0%)	3 (3%)
**Types of patients**^c^			
Outpatient	29 (12%)	19 (15%)	10 (8%)
Inpatient	204 (81%)	101 (79%)	103 (85%)
Only mild patients	21 (8%)	9 (7%)	12 (10%)
Only moderate patients	17 (7%)	6 (5%)	11 (9%)
Only severe patients	31 (12%)	17 (13%)	14 (12%)
Only critical patients	3 (1%)	0 (0%)	3 (2%)
Mixed patients	132 (53%)	69 (53%)	63 (52%)
Unclear	18 (7%)	10 (8%)	8 (7%)
**Treatments**			
Antimicrobials (antibiotics, antimalarials, antiparasitics)	52 (21%)	28 (22%)	24 (20%)
Antivirals	50 (20%)	20 (15%)	30 (25%)
Monoclonal antibodies	28 (11%)	14 (11%)	14 (12%)
Convalescent plasma	17 (7%)	11 (9%)	6 (5%)
Corticosteroids	11 (4%)	5 (4%)	6 (5%)
Interferons	9 (4%)	7 (5%)	2 (2%)
Other immunomodulators	9 (4%)	4 (3%)	5 (4%)
Supplements	9 (4%)	3 (2%)	6 (5%)
ATMP	7 (3%)	4 (3%)	3 (3%)
NSAIDs and anti-inflammatories	7 (3%)	5 (4%)	2 (2%)
Antithrombotic (antiplatelet, anticoagulant, thrombolytic drug)	6 (2%)	4 (3%)	2 (2%)
Kinase inhibitors	4 (2%)	3 (2%)	1 (1%)
Others	34 (14%)	20 (15%)	14 (12%)
Combinations	8 (3%)	2 (2%)	6 (5%)
**Funding**			
Public	101 (40%)	48 (37%)	53 (44%)
Mixed (public and private funding)	64 (25%)	41 (32%)	23 (19%)
Private	46 (18%)	26 (20%)	20 (17%)
No specific funding	27 (11%)	11 (8%)	16 (13%)
No information	13 (5%)	4 (3%)	9 (7%)

Percentages may not add up to 100% due to rounding

^a^One trial did not report the sample size (missing data)

^b^Most frequent countries of trials conducted in a single country

^c^The severity of the COVID-19 disease is based on the classification of the WHO Working Group on the Clinical Characterisation and Management of COVID-19 infection [24]

### Transparency indicators

#### Access to the trial documentation

**Fig. 2:**
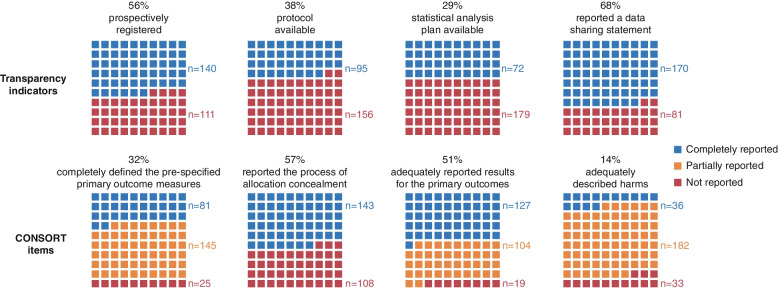
Reporting of transparency indicators and completeness of reporting of CONSORT items

A trial protocol was available for 38% (*n* = 95) of trials, and 4 (2%) were not in English (Fig. 2). A statistical analysis plan was accessible in only 29% of the trials (*n* = 72).

#### Trial registration

Overall, 239 trials (95%) were registered; about half of them were prospectively registered 140 (56%), and 94 (37%) were retrospectively registered of which 37/94 (39%) had a delay of more than 30 days between the trial start date and registration date (Fig. 2). For five trials (2%), we could not determine if the trial registry was prospectively posted due to unclear or missing information in the registry or report.

When the option for post-trial results was available (i.e., the registry offers the possibility to submit scientific and administrative information about the results of the trial that will be publicly displayed) (*n* = 164, 65%), only 27 (17%) posted their results in clinical trial registries (status last checked October 7, 2021). In addition, one trial provided a summary of results, despite the trial registry not providing an option to post the results.

#### Data sharing statement

Overall, 170 trials (68%) made a data sharing statement available in the report (Fig. 2). Of those, 155/170 trials (91%) stated their willingness to share data, and 14/170 trials (8%) stated that they were not willing to share their data, while one trial (1%) reported that they were undecided. Of the 155 trial reports stating an intention to share data, the authors reported that they would share data upon email request (*n* = 106, 68%) or in an online repository (*n* = 32, 21%), while 17 trials (11%) did not report how data would be made available. Of those, 79 trials (51%) defined the time frame for data sharing: 46 trials (30%) planned to share data after publication, 23 trials (15%) upon publication, 8 trials (5%) after completion of the trial, and 2 trials (1%) during the trial.

When a data sharing statement was reported in the registry (*n* = 177, 70%), we identified discrepancies between the registry and trial report for 58 trials: from data sharing willingness “no” or “undecided” in the registry to “yes” in the report (*n* = 54) and from “yes” in the registry to “no” in the report (*n* = 4). Overall, 42 trials had no information on data sharing in the registry but did include information in the corresponding trial report; 50 trials had information reported in the registry and no information in the corresponding report.

### Completeness of reporting

The results are detailed in Table 2, Fig. 2, and Additional file 1: Table S2. Overall, the completeness of reporting was low. Only 81 (32%) of the reports completely reported the pre-specified primary outcome; 206 (82%) described the methods used to generate the random allocation sequence and 143 (57%) the process of allocation concealment. Of the blinded trials (*n* = 111, 44%), less than half (*n* = 44, 40%) clearly described who was blinded and how. About half of the trials (*n* = 133, 53%) provided a complete description of the participant flow, either as a diagram or in text form.

**Table 2:** Completeness of reporting of CONSORT items and additional variables

Consort item	Checklist item	Complete reporting overall (***n*** = 251)	Complete reporting in preprints (***n*** = 130)	Complete reporting in journal publications (***n*** = 121)	Absolute risk reduction in % [95% confidence interval)
Section 6a, outcome	Completely defined pre-specified primary outcome measures, including how and when they were assessed	81 (32%)	39 (30%)	42 (35%)	5 [− 7–16]
Section 8a, sequence generation	Method used to generate the random allocation sequence	206 (82%)	107 (82%)	99 (82%)	0 [− 10–9]
Section 9, allocation concealment	Mechanism used to implement the random allocation sequence (e.g., sequentially numbered containers), describing any steps taken to conceal the sequence until interventions were assigned	143 (57%)	70 (54%)	73 (60%)	6 [− 6–19]
Section 11a/b, blinding	If done, who was blinded after assignment to interventions (e.g., participants, care providers, those assessing outcomes) and how	44/111 (40%)	23/61 (38%)	21/50 (42%)	4 [− 14–23]
Section 13a/b, participant flow	For each group, the numbers of participants who were randomly assigned, received intended treatment, and were analyzed for the primary outcome (including losses and exclusions with reasons)	133 (53%)	69 (53%)	64 (53%)	0 [− 13–12]
Section 17a, outcomes and estimation	For each primary outcome, results for each group and the estimated effect size and its precision (such as 95% confidence interval)	127 (51%)	67 (52%)	60 (50%)^a^	− 2 [− 14–11]
Section 19, harms^b^	All important harms or unintended effects in each group	36 (14%)	10 (8%)	26 (21%)^a^	13 [5–23]
Section 23, registration^c^	Registration number	231/239 (91%)	127/129 (99%)	104/110 (95%)	− 4 [− 9–1]
Overall	Overall CONSORT assessment	15 (6%)	5 (4%)	10 (8%)	4 [− 2–10]
**Additional items**					
Funding	Funding information	235 (94%)	125 (96%)	110 (91%)	− 5 [− 11–1]
Conflict of interest	Statement of conflicting interests	237 (94%)	125 (96%)	112 (93%)	− 3 [− 9–2]
Ethical approval	Statement of ethical approval	248 (99%)	130 (100 %)	118 (98%)	− 2 [− 5–0]

Percentages may not add up to 100% due to rounding

^a^One trial was not assessed, since only baseline data were presented. None of the pre-specified outcomes presented in the result section

^b^Harm defined as the totality of possible adverse consequences of an intervention and comprises the reporting of adverse events and serious adverse events [29]

^c^Twelve trials were not registered (12 peer-reviewed journal publications) and eight trials did not present the registration number in the report (six peer-reviewed journal publications and two preprints)

Regarding the study results, 127 trials (51%) reported the primary outcome(s) completely. Harm was adequately described in only 14% of the trials (*n* = 36). In particular, information regarding the mode of harm data collection (i.e., how data was collected) (*n* = 137, 55%) and the time frame of observation (*n* = 129, 51%) was insufficiently reported (Additional file 1: Table S2). Fifty-five trials (22%) did not report any results on harms, and most trials (*n* = 150, 60%) did not highlight whether harms resulted in withdrawals or trial discontinuations (Additional file 1: Table S2). Overall, only 6% (*n* = 15) completely reported the 10 most important CONSORT items.

Most trials reported information on funding (*n* = 235, 94%). Two hundred thirty seven (94%) disclosed information on conflicts of interest. All trials with the exception of three (99%) reported ethical approval.

For most items, reporting did not result in major differences between reports first published as a preprint and reports first published in a peer-reviewed journal (Table 2). The reporting of harm was slightly better in peer-reviewed journal publications (21% vs 8%; absolute risk difference [95% confidence interval (CI)] 13% [5-22].

### Reporting consistency (i.e., primary outcome switching)

Among all registered trials (*n* = 239), 212 trials (89%) identified their primary outcomes in the report and registry. Of those, 108 (51%) reported primary outcomes as pre-defined in the trial registry. Primary outcome switching between registered and published outcome(s) was identified in 104 trials (49%) (Table 3). Switches comprised completely changed primary outcome(s) (*n* = 39, 38%), reports that removed one or several primary outcomes (*n* = 19 18%), reports that added one or several primary outcomes (*n* = 9, 9%), and reports that added and removed one or several primary outcomes (*n* = 16, 15%). In addition, twenty-one trials (20%) changed the time frame or metric while the primary outcome variable stayed the same. Twelve trials (12%) had changes in time frames or metrics as well as added, removed, or changed primary outcome(s). Overall, 16 trials (15%) justified the primary outcome switching in the report.

**Table 3:** Primary outcome switching between the registry and report

Outcome switch	Example	***N*** = 104 (%)
**Added primary outcome(s)**	Registry: 1. Time to clinical improvement Report: 1. Death 2. Time to clinical improvement	9 (9%)
**Removed primary outcome(s)**	Registry: 1. Time and rate of temperature return to normal 2. Time and rate of improvement of respiratory symptoms and signs 3. Time and rate of change to negative COVID-19 nucleic acid test 4. Rate of mild/moderate type to severe type, rate of severe type to critical type Report: 1. Rate of nucleic acid negativity conversion of SARS-CoV-2 2. Negativity conversion time	19 (18%)
**Added and removed primary outcome(s)**	Registry: 1. Hospitalization days 2. Need for mechanical ventilation 3. Condition of discharge (death or recovery) Report: 1. Improvement in the rate of ICU admissions 2. Intubation/mechanical ventilation 3. Mortality 28 days	16 (15%)
**Changed primary outcome(s)**	Registry: 1. Time to improvement Report: 2. Clinical status	39 (38%)
**Time frame or metric different**	Registry: 1. Change in clinical status of subjects at day 7 Report: 2. Change in clinical status of subjects at day 15	21 (20%)^a^

Percentages may not add up to 100% due to rounding

^a^Twelve additional trials comprised both, changes in time frames or metrics, as well as added, removed, or changed primary outcome(s). Those trials were counted only a single time within the added, removed, or changed outcome switching domain

### Comparison between preprint report and subsequent peer-reviewed journal publication

#### Reports identification

Of 130 preprints included in our analysis, we identified 78 corresponding subsequent peer-reviewed journal publications. The median time between preprint and publication in a peer-reviewed journal was 94 days (IQR: 55–168) (range: 5–505). The protocol and statistical analysis plan were added in 14 (18%) and 11 (14%) peer-reviewed journal publications, respectively. However, the protocol and the statistical analysis plan were removed in 5 (6%) and 3 (4%) reports, respectively.

#### Differences in the completeness of reporting and primary outcome switching

**Fig. 3:**
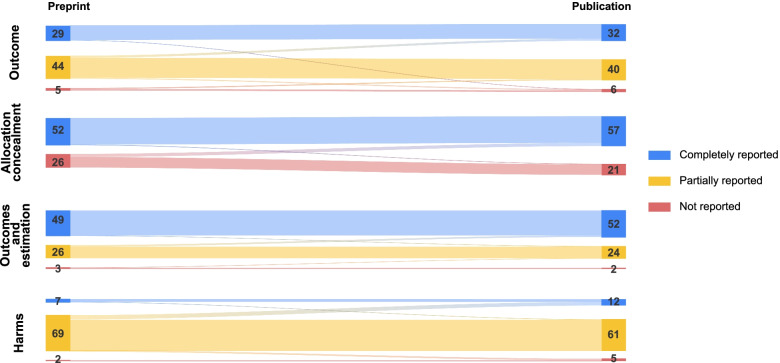
Differences in the completeness of reporting between preprint reports and subsequent peer-reviewed journal publications

The detailed differences between preprint and peer-reviewed journal publication are described in Table 4, Fig. 3, and Additional file 1: S3 Table. Information regarding the completeness of reporting after the journal peer review process was rarely added to the report: allocation concealment (*n* = 6, 8%), the persons who were blinded (*n* = 5, 6%), the mode of harm data collection (*n* = 6, 8%), and the time frame of harm surveillance (*n* = 6, 8%). Information that was removed from the preprint in the peer-reviewed journal publication included the mode of harm data collection (*n* = 4, 5%), the description of the primary outcome (*n* = 3, 4%), or the registration number (*n* = 3, 4%). Overall, 41 trials (53%) changed at least one CONSORT sub-item from the preprint to the peer-reviewed journal publication. Only two (3%) of the trials had changed their overall CONSORT assessment from partially to completely reported.

**Table 4:** Changes in CONSORT sub-items between preprint and peer-reviewed journal publication (*n* = 78)

Consort item	Reported, no change	Not reported, no change	Added	Removed	Not applicable
**Section 6a**					
Clear primary outcome	70 (90%)	3 (4%)	2 (3%)	3 (4%)	0 (0%)
Variable of interest	69 (88%)	1 (1%)	0 (0%)	0 (0%)	8 (10%)^a^
How the outcome was assessed	62 (79%)	8 (10%)	0 (0%)	0 (0%)	8 (10%)^a^
The analysis metric	70 (90%)	0 (0%)	0 (0%)	0 (0%)	8 (10%)^a^
The summary measure for each study group	58 (74%)	9 (12%)	3 (4%)	0 (0%)	8 (10%)^a^
Time point of interest for analysis	63 (81%)	4 (5%)	2 (3%)	1 (1%)	8 (10%)^a^
Who assessed the outcome	33 (42%)	32 (41%)	4 (5%)	1 (1%)	8 (10%)^a^
**Sections 8a and 9**					
Method of sequence generation	71 (91%)	4 (5%)	3 (4%)	0 (0%)	0 (0%)
Mechanism allocation concealment	51 (65%)	20 (26%)	6 (8%)	1 (1%)	0 (0%)
**Section 11a/b**					
Who was blinded	25 (32%)	6 (8%)	5 (6%)	0 (0%)	42 (54%)^b^
How the blinding was performed	31 (40%)	4 (5%)	1 (1%)	0 (0%)	42 (54%)^b^
Similarities of the characteristics of the interventions	18 (23%)	11 (14%)	4 (5%)	1 (1%)	44 (56%)^b^
**Section 13 a/b**					
Flow chart	66 (85%)	8 (10%)	4 (5%)	0 (0%)	0 (0%)
Participants randomized	75 (96%)	2 (3%)	1 (1%)	0 (0%)	0 (0%)
Participants who received treatment	68 (88%)	8 (10%)	2 (3%)	0 (0%)	0 (0%)
Participants lost to follow-up	77 (99%)	1 (1%)	0 (0%)	0 (0%)	0 (0%)
Participants who discontinued intervention	51 (65%)	23 (29%)	3 (4%)	1 (1%)	0 (0%)
Participants analyzed	76 (97%)	2 (3%)	0 (0%)	0 (0%)	0 (0%)
**Section 17a**					
Result	74 (95%)	2 (3%)	1 (1%)	1 (1%)	0 (0%)
Difference in estimated effect	49 (63%)	24 (31%)	4 (5%)	1 (1%)	0 (0%)
Precision of the estimated effect	48 (62%)	1 (1%)	0 (0%)	0 (0%)	29 (36%)^c^
**Section 19**					
List of harms addressed	43 (55%)	29 (37%)	4 (5%)	2 (3%)	0 (0%)
Mode of data collection	37 (47%)	31 (40%)	6 (8%)	4 (5%)	0 (0%)
Time frame of surveillance	35 (45%)	33 (42%)	6 (8%)	4 (5%)	0 (0%)
Person responsible making attribution	30 (38%)	39 (50%)	5 (6%)	4 (5%)	0 (0%)
Participant withdrawals due to harm	32 (41%)	43 (55%)	1 (1%)	2 (3%)	0 (0%)
Results of each harm type	67 (86%)	8 (10%)	2 (3%)	1 (1%)	0 (0%)
**Section 23**					
Registration number	75 (96%)	0 (0%)	0 (0%)	3 (4%)	0 (0%)

Percentages may not add up to 100% due to rounding

^a^Eight publications did not clearly specify the primary outcome (either in preprint or journal publication). Due to that the full assessment of CONSORT, section 6a was (items 2–7) was not possible

^b^Forty-two trials were unblinded and could not be assessed. Two additional trials did not use a placebo control and could not be assessed for the item similar characteristics of the intervention (CONSORT section 11a/b, item 3)

^c^Twenty-nine trials did not present the difference in estimated effect measure (CONSORT section 17a, item 2). Precision was therefore not assessable for 29 trials (item 3)

Of the 78 assessed trials, nine trials could not be compared for the differences in primary outcome switching due to the missing definition of the primary outcome in the pre-print (*n* = 3), peer-reviewed journal publication (*n* = 2), or both (*n* = 4). There was no change in primary outcome switching between preprint and peer-reviewed journal publication. Only one trial added a justification for primary outcome switching in the peer-reviewed journal publication.

## Discussion

This study provides a detailed description of transparency and completeness of reporting for all randomized controlled trials assessing pharmacological treatment of COVID-19 published up to May 2021. Most of the trials identified were 2-arm trials assessing repurposed treatments for hospitalized patients. The sample size was small with about 40% being single-center trials.

Transparency indicators were suboptimal with less than half providing access to a protocol and one-third to a statistical analysis plan, and very few posted trial results on registries. In contrast, most trials were registered, although registration was often retrospective which could be related to the urgency and exigency of the situation where the start of inclusion needed to be as quick as possible to be in line with the COVID-19 waves. Interestingly, a large number of trials provided a data sharing statement and indicated their willingness to share. This could be the consequence of the change in the policy of the International Committee of Medical Journal Editors (ICMJE) which requests since July 2018 that all reports contain a data sharing statement and that clinical trials enrolling participants since January 2019 include a data sharing plan in the trial’s registration [29]. Nevertheless, we cannot extrapolate that the investigator stating their willingness to share will actually share their data [30, 31]. A study found that among the preprint articles about COVID-19 reporting data availability, raw data were actually available for less than half of these articles [32].

Despite the development of the Consolidated Standards of Reporting Trials (CONSORT), research waste due to incomplete reporting is still substantial in both preprint and peer-reviewed publication [33]. The reporting of harm was particularly poor. An extension of the CONSORT statement was developed in 2004 [26]. However, inadequate reporting remains prevalent [34]. An update of this extension has been planned.

Our results also identified a high prevalence of switch in outcomes in COVID-19 trials. Several studies have highlighted discrepancies between outcomes planned in the protocol/registry and reported outcomes in the publications which is suspect of selective reporting of outcomes [35, 36]. This high prevalence could be explained by the novelty of the disease and the rapid increase in knowledge over time which may have required important changes to the protocol. Nevertheless, the lack of transparency related to these changes is concerning. Furthermore, despite this evidence and the recommendation to compare outcomes to the outcomes in the register, this is rarely done by peer reviewers [37].

Worth mentioning, while no study was retracted at the time of our search, the COVID-NMA initiative identified that five of the included reports have been retracted since May 2021 (two preprints that were subsequently published, two preprints that were never published, and one peer-reviewed publication) (Additional file 1: Table S2).

The identification of the pairs of preprint-peer-reviewed publications allows for exploring the impact of the journal peer review process on the content of the manuscript. When comparing the completeness of reporting between those pairs, we did not identify major improvement after the journal peer review process for most items. Despite a considerable delay (a median of 94 days) between the publication of the results on a preprint platform and the publication in a peer-reviewed journal, the journal peer review process had a low impact on transparency, completeness, and accuracy of reporting.

### Comparison with other studies

Our results are consistent with other studies assessing reporting characteristics of RCTs. Before the COVID-19 pandemic, an analysis of more than 20,000 RCTs included in Cochrane reviews showed important deficiencies in reporting which is a strong barrier to risk of bias assessment and the extraction of outcomes needed to conduct systematic reviews and meta-analyses [38]. More recently, studies assessing reporting and design of COVID-19 trials at an early stage of the pandemic highlighted the limitation in design and reporting practices [12, 39]. In contrast, Jung et al. concluded in a research letter that the reporting of RCTs of COVID-19 pharmacological treatments was adequate for most items [40].

Our results are also consistent with studies exploring the consistencies between sources. Shi et al. found no major differences in the content of 47 clinical studies posted as preprints from June 2019 to August 2020 and subsequently published in high-impact journals [41]. A study comparing preprint and published articles at the initial phase of the COVID-19 pandemic using automatic and manual annotation at the beginning of the pandemic found very modest changes in the content between the two sources [42]. Bero et al. found that the reporting of outcome and spin was similar in 67 pairs of preprint-related journal articles of interventional and observational studies of COVID-19 interventions published between March and October 2020 [18]. Oikonomidi et al. assessed the consistencies between preprint versions and between preprint and related journal publication in a sample of observational and interventional COVID-19 studies published up to August 2020 and found important changes in the study results in one-fifth of the reports and change in conclusion in one-fourth [20].

### Strengths and limitations

Our study extensively assessed the reporting characteristics of all COVID-19 RCTs assessing pharmacological treatments published as preprint or peer-reviewed journal article during the first 17 months of the pandemic. Our sample has been included in a large living network meta-analysis and is comprehensive. Furthermore, we assessed the various dimensions of transparency, reporting, and consistency between reports and registry records. Finally, to our knowledge, it is the largest study comparing preprint and subsequent publications in the field of COVID-19.

Our study has some limitations. First, we focused on randomized controlled trials and cannot extrapolate to other study designs. Nevertheless, RCTs are considered the gold standard for therapeutic evaluation. Second, we cannot exclude the possibility that we missed some preprint servers as these developed rapidly over time. However, the search developed by the *L·OVE COVID-19 platform* searches most preprint servers*.* Finally, most trials were assessed by a single researcher, despite a random sample being extracted in duplicate and showing good reproducibility with a kappa coefficient of 0.87 (95% CI, 0.83–0.92).

### Implications

Our results have important implications. There is an urgent need for high-quality evidence to guide the management of COVID-19 patients. It is consequently essential to improve reporting and transparency and increase adherence to the CONSORT statement. As part of the COVID-NMA living review, we are already systematically contacting investigators to request the missing data. Furthermore, we plan to inform investigators of their results in terms of reporting and transparency to help them improve the content of their reports.

The publication of results on preprint servers became an essential means of communication. It was adopted considerably by the research community during this pandemic mainly because it shortened delays between the production of reports and their dissemination to the community. In our sample, half of the trials decided to communicate first through preprint. Overall, it reduced the delay of accessing results by a median of 3 months. Some researchers, decision-makers, funders, and editors raised concerns related to the risk of disseminating reports that were not peer-reviewed [8, 13]. However, our results do not support the hypothesis that peer-reviewed journal publications are of better reporting quality compared to preprints. We found no difference in terms of transparency and reporting between the preprint and the peer-reviewed report.

Finally, our results question the publication process and role of the journal peer review process in improving reporting and transparency. Our results are consistent with other studies comparing the completeness of reporting of the submitted report to the published report focusing on RCTs [28]. We need to develop specific interventions and tools to increase the detection and improvement of reporting in publications. Some tools such as the CobPeer tool have been proposed and evaluated [27]. Other interventions targeting preprints could be useful to inform trialists of reporting deficiencies and help them improve their report prior to publications. Public, open, post-publication peer reviews such as PubPeer are also essential and were instrumental during the pandemic to detect errors, low-quality studies, and misleading interpretations [43, 44]. Some authors proposed new approaches to speed up the scientific correction process and to improve the science communication through open science [8, 43].

## Conclusions

In conclusion, the lack of transparency, completeness, and consistency of reporting is an important barrier to trust, interpretation, and synthesis in COVID-19 clinical trials. Peer-reviewed publications were not better than preprints in this regard. Furthermore, the journal peer review process failed to improve the deficiency in reporting.

Trial authors as well as editors and funders must apply higher standards of methodological rigor and transparency to ensure the generation of the highest level of evidence to inform decision-making and curb the pandemic.

## Supplementary Information

**Additional file 1: Table S1**. Search strategy. **Table S2**. Completeness of reporting of CONSORT sub-items. **Table S3**. Changes in CONSORT items between preprint and peer-reviewed journal publication. **Table S4**. Description of studies that were retracted or removed after the search date.

**Additional file 2.** List of excluded trials.

## Data Availability

The datasets used and analyzed during the current study are available from the corresponding author upon reasonable request.

## Abbreviations

ATMP: Advanced therapy medicinal products
RCT: Randomized controlled trial
