# Supplementary material for: Transparency and reporting characteristics of COVID-19 randomized controlled trials
Source: BMC Med. 2022 Sep 26;20:363. doi: 10.1186/s12916-022-02567-y (PMC9510360; doi:10.1186/s12916-022-02567-y)
Supplement: Supplementary file 1 — Additional file 1: Table S1. Search strategy. Table S2. Completeness of reporting of CONSORT sub-items. Table S3. Changes in CONSORT items between preprint and peer-reviewed journal publication. Table S4. Description of studies that were retracted or removed after the search date. [file 12916_2022_2567_MOESM1_ESM.docx]

**Additional file 1**

**S1 Table Search strategy**

The search strategy was developed in the context of the COVID-NMA initiative for the living systematic review of all treatment interventions for COVID-19 with the support of an information specialist. The search strategy evolved over time after the availability of high-quality secondary sources: 1) the Epistemonikos L·OVE COVID-19 platform and 2) The Cochrane Covid-19 Study Register. Below we describe our initial search strategy and secondary sources.

**First Period of search**

Up to September 2020, we relied on the following sources:

| **PubMed (MEDLINE)** | (2019 nCoV[tiab] OR 2019nCoV[tiab] OR corona virus[tiab] OR corona viruses[tiab] OR coronavirus[tiab] OR coronaviruses[tiab] OR COVID[tiab] OR COVID19[tiab] OR nCov 2019[tiab] OR SARSCoV2[tiab] OR SARS CoV-2[tiab] OR SARSCoV2[tiab] OR SARSCoV-2[tiab] OR "COVID-19"[Mesh] OR "COVID-19 Testing"[Mesh] OR "COVID-19 Vaccines"[Mesh] OR "Coronavirus"[Mesh:NoExp] OR "SARS-CoV-2"[Mesh] OR "COVID-19"[nm] OR "COVID-19 drug treatment"[nm] OR "COVID-19 diagnostic testing"[nm] OR "COVID-19 serotherapy"[nm] OR "COVID-19 vaccine"[nm] OR "LAMP assay"[nm] OR "severe acute respiratory syndrome coronavirus 2"[nm] OR "spike protein, SARSCoV-2"[nm]) NOT ("animals"[mh] NOT "humans"[mh]) NOT (editorial[pt] OR newspaper article[pt]) |
| --- | --- |
| **Embase.com** | ((('coronaviridae'/de OR 'coronavirinae'/de OR 'coronaviridae infection'/de OR 'coronavirus disease 2019'/exp OR 'coronavirus infection'/de OR 'SARS-related coronavirus'/de OR 'Severe acute respiratory syndrome coronavirus 2'/exp OR '2019 nCoV':ti,ab,kw OR 2019nCoV:ti,ab,kw OR ((corona* OR corono*) NEAR/1 (virus* OR viral* OR virinae*)):ti,ab,kw OR coronavir*:ti,ab,kw OR coronovir*:ti,ab,kw OR COVID:ti,ab,kw OR COVID19:ti,ab,kw OR HCoV*:ti,ab,kw OR 'nCov 2019':ti,ab,kw OR 'SARS CoV2':ti,ab,kw OR 'SARS CoV 2':ti,ab,kw OR SARSCoV2:ti,ab,kw OR 'SARSCoV 2':ti,ab,kw) NOT (('animal experiment'/de OR 'animal'/exp) NOT ('human'/exp OR 'human experiment'/de))) NOT 'editorial'/it) NOT ([medline]/lim OR [pubmed-not-medline]/lim) AND [1-12-2019]/sd |
| **Cochrane Central Register of Controlled trials (CENTRAL)** | ("2019 nCoV" OR 2019nCoV OR "corona virus*" OR coronavirus* OR COVID OR COVID19 OR "nCov 2019" OR "SARS-CoV2" OR "SARS CoV-2" OR SARSCoV2 OR "SARSCoV-2"):TI,AB AND CENTRAL:TARGET 2 Coronavirus:MH AND CENTRAL:TARGET 3 Coronavirus:EH AND CENTRAL:TARGET 4 #1 OR #2 OR #3 5 2019 TO 2021:YR AND CENTRAL:TARGET 6 #5 AND #4 7 INSEGMENT 8 #6 NOT #7 |
| **MedRχiv** | A curated list of records for COVID-19 and SARS-CoV-2 is available at https://connect.biorxiv.org/relate/ content/181. Note that this list also includes sources listed in bioRχiv, but we only screened the sources published on MedRχiv. |
| **Chinaχiv** | **Searched** up to 7 April 2020 |

- We also searched ICTRP Search Portal and Clinicaltrials.gov
- We also searched The Cochrane Covid-19 Study Register used as quality control and Epistemonikos L·OVE COVID-19 platform from June 2020.

**Second Period (from September 2020)**

Since September 2020, we relied on the following sources:

*1 The* Living OVerview of Evidence (*L-OVE) platform*

Details related to the search performed by this platform and the process is available here https://app.iloveevidence.com/loves/5e6fdb9669c00e4ac072701d?population=5e7fce7e3d05156b5f5e032a&intervention_variable=603b9fe03d05151f35cf13dc&section=methods&classification=all.

In brief, the Living OVerview of Evidence (L·OVE) was built, and is maintained, by systematic searches in multiple databases, trial registries and preprint servers. The following sources are regularly searched:

- Pubmed/medline (updated several times a day)
- EMBASE (updated weekly)
- CINAHL (updated weekly)
- PsycINFO (updated weekly)
- LILACS (Latin American & Caribbean Health Sciences Literature) (updated weekly)
- Wanfang Database (updated every 2 weeks)
- CBM - Chinese Biomedical Literature Database (updated every 2 weeks)
- CNKI - Chinese National Knowledge Infrastructure (updated every 2 weeks)
- VIP - Chinese Scientific Journal Database (updated every 2 weeks)
- IRIS (WHO Institutional Repository for Information Sharing) (updated weekly)
- IRIS PAHO (PAHO Institutional Repository for Information Sharing)) (updated weekly)
- IBECS - Índice Bibliográfico Español en Ciencias de la Salud (Spanish Bibliographic Index on Health Sciences) (updated weekly)
- Microsoft Academic (last searched: 23 August 2021)
- ICTRP Search Portal (updated daily)
- Clinicaltrials.gov (updated daily)
- ISRCTN registry (updated daily)
- Chinese Clinical Trial Registry (updated daily)
- IRCT - Iranian Registry of Clinical Trials (updated daily)
- EU Clinical Trials Register: Clinical trials for covid-19 (updated daily)
- NIPH Clinical Trials Search (Japan) - Japan Primary Registries Network (JPRN) (JapicCTI, JMACCT CTR, jRCT, UMIN CTR) (updated daily, via ICTRP search portal)
- UMIN-CTR - UMIN Clinical Trials Registry (updated daily, via ICTRP search portal)
- JRCT - Japan Registry of Clinical Trials (updated daily, via ICTRP search portal)
- JAPIC Clinical Trials Information (updated daily, via ICTRP search portal)
- Clinical Research Information Service (CRiS), Republic of Korea (updated daily, via ICTRP search portal)
- ANZCTR - Australian New Zealand Clinical Trials Registry (updated daily, via ICTRP search portal)
- ReBec - Brazilian Clinical Trials Registry (updated daily, via ICTRP search portal)
- CTRI - Clinical Trials Registry - India (updated daily, via ICTRP search portal)
- RPCEC - Cuban Public Registry of Clinical Trials (updated daily, via ICTRP search portal)
- DRKS - German Clinical Trials Register (updated daily, via ICTRP search portal)
- LBCTR - Lebanese Clinical Trials Registry (updated daily, via ICTRP search portal)
- TCTR - Thai Clinical Trials Registry (updated daily, via ICTRP search portal)
- NTR - The Netherlands National Trial Register (updated daily, via ICTRP search portal)
- PACTR - Pan African Clinical Trial Registry (updated daily, via ICTRP search portal)
- REPEC - Peruvian Clinical Trial Registry (updated daily, via ICTRP search portal)
- SLCTR - Sri Lanka Clinical Trials Registry (updated daily, via ICTRP search portal)
- medRxiv (updated several times a day)
- bioRxiv (updated several times a day)
- SSRN Preprints (updated several times a day)
- ChinaXiv (updated every 2 weeks)
- SciELO Preprints (updated weekly)
- Research Square (updated daily)

*2) The Cochrane Covid-19 Study Register*

Details related to the search performed by this register and the process are described here: https://community.cochrane.org/about-covid-19-study-register. It is a specialised register built within the Cochrane Register of Studies (CRS) and is maintained by Cochrane Information Specialists. The register contains study reports from several sources, including:

- daily searches of PubMed
- daily searches of ClinicalTrials.gov
- weekly searches of Embase.com
- weekly searches of the WHO International Clinical Trials Registry Platform (ICTRP)
- weekly searches of medRxiv
- monthly searches of the Cochrane Central Register of Controlled Trials (CENTRAL)

*3) Retraction Watch* We also searched the Retraction Watch Database for retracted studies (retractionwatch.com/retracted-coronavirus-covid-19-papers/).

**S2 Table Completeness of reporting of CONSORT sub-items (N=251)**

| **CONSORT item** | **Checklist item** | **Reported  N (%)** |
| --- | --- | --- |
| **Section 6a** |  |  |
| Clear primary outcome | Was the primary outcome(s) clearly identified (e.g., the primary/main outcome was pain)? | 226 (90%) |
| Variable of interest | The variable of interest (e.g., pain, all-cause mortality) | 223 (89%) |
| How the outcome was assessed | How the outcome was assessed (e.g., VAS, Beck Depression Inventory score, pain scale) | 194 (77%) |
| The analysis metric | The analysis metric (e.g., change from baseline, final value, time to event) | 221 (88%) |
| The summary measure for each study group | The summary measure for each study group (e.g., mean, proportion with score > 2) | 190 (76%) |
| Time point of interest for analysis | Time point of interest for analysis (e.g., 3 months)**NA if survival analysis* | 201 (80%) |
| Who assessed the outcome | Who assessed the outcome (e.g., the patient, doctor, nurse, caregiver, other) | 98 (39%) |
| **Section 8a and 9** |  |  |
| Method of sequence generation | Method used to generate the random allocation sequence | 206 (82%) |
| Mechanism allocation concealment | Mechanism used to implement the random allocation sequence (e.g., sequentially numbered containers), describing any steps taken to conceal the sequence until interventions were assigned | 143 (57%) |
| **Section 11a/b (N=111)** |  |  |
| Who was blinded | Was the study blinded? | 78 (70%) |
| How the blinding was performed | Who (i.e., participants, healthcare providers, data collectors, outcome adjudicators, and data analysts) was blinded to treatment assignments? | 89 (80%) |
| Similarities of the characteristics of the interventions | How was the blinding performed? (e.g., used of placebo, intervention by physician unaware of the study) | 50 (45%) |

| **CONSORT item** | **Checklist item** | **Reported  N (%)** |
| --- | --- | --- |
| **Section 13 a/b** |  |  |
| Flow chart | Did the authors report a flow chart | 211 (84%) |
| Participants randomized | Number of participants randomized in each group | 240 (96%) |
| Participants who received treatment | Number of participants who received intended treatment in each group | 209 (83%) |
| Participants lost to follow-up | Number of participants lost to follow-up with reasons in each group | 242 (96%) |
| Participants who discontinued intervention | Number of participants who discontinued intervention with reasons in each group | 156 (62%) |
| Participants analyzed | Number of participants analyzed for the primary outcome in each group | 242 (96%) |
| **Section 17a** |  |  |
| Result* | Result in each group (mean (SD) or number of events/N) | 230 (92%) |
| Difference in estimated effect | Difference in estimated effect between groups (e.g., odds ratio (OR), risk ratio (RR), risk difference (RD), hazard ratio (HR), difference in median survival time, mean difference (MD)) | 129 (52%) |
| Precision | Precision for difference between groups (e.g., 95% CI) | 128 (51%) |
| **Section 19** |  |  |
| List of harms addressed | How harms-related information was collected (List of harm related events that were addressed) | 135 (54%) |
| Mode of data collection | Mode of data collection (Full description of methods used to collect the harm related information) | 114 (45%) |
| Time frame of surveillance | Timing (description of time frame of surveillance) | 122 (49%) |
| Person responsible making attribution | Attribution methods (Person responsible making attribution disclosed and whether blinding was used) | 99 (39%) |
| Participant withdrawals due to harm* | For each group, participant withdrawals due to harm | 100 (40%) |
| Results of each harm type* | Results in each group for each harms type with denominator (mean [SD] or number of events/N) | 195 (78%) |
| **Section 23 (N=239)** |  |  |
| Registration number | The registration number | 231 (97%) |

**S2 Table (continued)**.

* One trial not assessed, since only baseline data were presented. None of the pre-specified outcomes presented in the result section.

**S3 Table Changes in CONSORT items between preprint and peer-reviewed journal publication (n=78)**

| **Consort item** | **Completely reported,**  **not changed** | **Partially reported, not changed** | **Not reported, not changed** | **Changed to completely reported from partial** | **Changed to completely reported from not reported** | **Changed to partially reported from not reported** | **Changed to partially reported from complete** | **Changed to not reported from complete** | **Changed to not reported from partial** |
| --- | --- | --- | --- | --- | --- | --- | --- | --- | --- |
| Section 6a, outcome | 28 (36%) | 38 (49%) | 3 (4%) | 4 (5%) | 0 (0%) | 2 (3%) | 0 (0%) | 1 (1%) | 2 (3%) |
| Section 8a, sequence generation | 71(91%) | - | 4 (5%) | - | 3 (4%) | - | - | 0 (0%) | - |
| Section 9, allocation concealment | 51 (65%) | - | 20 (26%) | - | 6 (8%) | - | - | 1 (1%) | - |
| Section 11b, blinding***** | 16 (21%) | 12 (15%) | 1 (1%) | 6 (8%) | 0 (0%) | 0 (0%) | 1 (1%) | 0 (0%) | 0 (0%) |
| Section 13a/b, participant flow | 44 (56%) | 28 (36%) | 0 (0%) | 5 (6%) | 0 (0%) | 0 (0%) | 1 (1%) | 0 (0%) | 0 (0%) |
| Section 17a, outcomes and estimation | 48 (62%) | 22 (28%) | 2 (3%) | 4 (5%) | 0 (0%) | 1 (1%) | 1 (1%) | 0 (0%) | 0 (0%) |
| Section 19, harms | 6 (8%) | 60 (77%) | 2 (3%) | 6 (8%) | 0 (0%) | 0 (0%) | 1 (1%) | 0 (0%) | 3 (4%) |
| Section 23, registration | 75 (96%) | - | 0 (0%) | - | 3 (4%) | - | - | 0 (0%) | - |
| Overall | 5 (6%) | 71 (91%) | 0 (0%) | 2 (3%) | 0 (0%) | 0 (0%) | 0 (0%) | 0 (0%) | 0 (0%) |

Percentages may not add up to 100% due to rounding.
* 42 trials were unblinded

**S4 Table Description of studies that were retracted or removed after the search date. These studies are included in our sample as they were available at the time of our search**

| **Reference** | **Treatment** | **Registration number** | **Retracted date** | **Protocol reported** | **Reason for retraction** | **Link to retraction note** | **CONSORT overall assessment** |
| --- | --- | --- | --- | --- | --- | --- | --- |
| Dabbous HM, Arch Virol, 2021 | Favipiravir | NCT04351295 | 2021-11-22 | No | Methodological concerns | https://www.ncbi.nlm.nih.gov/pmc/articles/PMC8608235/ | Partially reported |
| Dabbous HM, Sci Rep, 2021 | Favipiravir | NCT04349241 | 2021-09-18 | No | Methodological concerns | https://www.ncbi.nlm.nih.gov/pmc/articles/PMC8462367/ | Partially reported |
| Elgazzar A, ResearchSquare, 2021 | Ivermectin | NCT04668469 | 2021-07-14 | No | Potential fabrication and plagiarism | https://grftr.news/why-was-a-major-study-on-ivermectin-for-covid-19-just-retracted/ | Partially reported |
| Pott-Junior H, Toxicology Reports, 2021 | Ivermectin | NCT04431466 | 2022-05-02 | No | Methodological concerns and insufficient reporting | https://www.ncbi.nlm.nih.gov/pmc/articles/PMC9060540/ | Partially reported |
| Youssef J, SSRN, 2021 | Aviptadil | NCT04311697 | Not reported | No | Not reported | https://papers.ssrn.com/sol3/papers.cfm?abstract_id=3794262 | Partially reported |
